# Indicadores Socioeconômicos e Mortalidade por Insuficiência Cardíaca: Parâmetros Indissociáveis?

**DOI:** 10.36660/abc.20210826

**Published:** 2021-11-01

**Affiliations:** 1 Faculdade de Medicina de Jundiaí Group of Investigation on Multimorbidity and Mental Health in Aging Jundiaí SP Brasil Group of Investigation on Multimorbidity and Mental Health in Aging (GIMMA) - Faculdade de Medicina de Jundiaí, Jundiaí, SP - Brasil; 2 Hospital Samaritano de São Paulo São Paulo SP Brasil Hospital Samaritano de São Paulo, São Paulo, SP - Brasil; 3 Universidade de Pernambuco Pronto Socorro Cardiológico de Pernambuco Recife PE Brasil PROCAPE (Pronto Socorro Cardiológico de Pernambuco) - Universidade de Pernambuco, Recife, PE - Brasil; 4 Centro Cardiológico Ovídio Montenegro Recife PE Brasil Centro Cardiológico Ovídio Montenegro, Recife, PE - Brasil

**Keywords:** Doenças Cardiovasculares, Insuficiência Cardíaca, Mortalidade, Fatores de Risco, Classe Social, Desenvolvimento Humano

As doenças cardiovasculares (DCV) continuam sendo a principal causa de morte, sendo responsável por aproximadamente um terço dos óbitos no mundo. Em junho de 2021, a Organização Mundial da Saúde (OMS) reforçou sua preocupação com o impacto causado pelas DCV nos países de baixa ou média renda, onde ocorrem mais de três quartos de seus óbitos.^1^ Via final comum das cardiopatias, a insuficiência cardíaca (IC) se apresenta como pandemia global fora de controle, com prevalência em crescimento, como consequência de fatores como o envelhecimento da população, maior presença de fatores de risco cardiovasculares, como obesidade, sedentarismo ou diabetes mellitus, mesmo com avanços terapêuticos que reduzem a mortalidade.^2^

A relação entre piores condições socioeconômicas e maior mortalidade por IC parece ter sido bem estabelecida nos últimos anos em diferentes populações,^3–5^ justificada em parte pelo pior acesso a métodos diagnósticos e tratamento farmacológico. Entretanto, esta relação é mais confusa em países de baixa e média renda, onde as variáveis clínicas, demográficas e socioeconômicas explicam pouco sobre a variabilidade entre as taxas de mortalidade em um ano por IC entre regiões da África, Índia, Sudeste Asiático, Oriente Médio, América do Sul e China, conforme observado no *INTER-CHF Prospective Cohort Study.*^6^

Nas últimas décadas, o Brasil apresentou uma queda gradual da desigualdade, medida pelo coeficiente de Gini – principalmente a partir de meados dos anos 90 e atingindo seu mínimo em 2010^7^ –, assim como uma melhora progressiva Índice de Desenvolvimento Humano (IDH) e seu equivalente regionalizado por município (IDHM), cujos valores retratam três dimensões básicas do desenvolvimento humano: longevidade, educação e renda.^8^ Na mesma linha, a publicação de Malta et al.,^9^ trouxe dados recentes que confirmam que a taxa de mortalidade cardiovascular ajustada também apresentou queda no Brasil nos últimos anos, embora já chamasse atenção uma heterogeneidade entre as Unidades da Federação (UF). Eis que o estudo dessa edição do ABC^10^ se propõe a analisar a relação entre a evolução temporal do desenvolvimento humano e as taxas de mortalidade por insuficiência cardíaca nas diferentes regiões geográficas do Brasil, trazendo importantes luzes ao assunto.

De acordo com esse artigo,^10^ a redução da mortalidade por IC de fato ocorreu em todas as UF. Porém, embora a redução da mortalidade nos estados onde houve menor incremento do IDHM (RJ, DF, SP, RS, SC e ES) tenha sido maior, todos esses estados já apresentavam alto IDMH (> 0,7). Por outro lado, os autores observam que o IDMH também melhorou em todas as UFs. E os estados que apresentaram os maiores incrementos do IDMH (TO, MA, PI, PB, AL e BA), porém permanecendo com índices inferiores a 0,7, tiveram menores reduções de mortalidade por IC. Esses dados sugerem fortemente, portanto, que para alcançar grandes reduções na taxa de mortalidade por IC, “mais importante que o grau de incremento do IDHM é o nível final que ele alcança” – conforme dito pelos autores.

A mortalidade de uma doença crônica não contagiosa como a insuficiência cardíaca e indicadores socioeconômicos, ao que parece, não são parâmetros tão dissonantes assim. Ao contrário, pode ser que essas duas linhas se encontrem com o passar do tempo, caso haja uma redução das desigualdades e todas as regiões alcancem bons índices de desenvolvimento (IDHM >0,7). Ou até mesmo essas linhas se afastem ainda mais, caso persista a piora dos indicadores em saúde no país que temos observado nos últimos anos, com aumento da pobreza, cortes em políticas sociais e congelamento dos recursos da saúde produzidos pela Emenda Constitucional no. 95, já citados recentemente.^9,11^ E embora o IDH represente apenas uma visão parcial do status socioeconômico de uma população, não se podendo avaliar diretamente sobre a relação de desigualdade e mortalidade por IC, destacamos que é razoável inferir que variações importantes de IDH entre as regiões permitem conhecer focos de desigualdade no território nacional. Um IDH baixo reflete, na esmagadora maioria das vezes, uma população pobre e com déficit educacional importante, o que leva a maiores dificuldades de se entender, adquirir e aderir a um tratamento médico complexo como o da IC.

O artigo reforça a impressão de que boas condições de vida (socioeconômicas e educacionais) parecem estar intrinsecamente ligadas a melhores desfechos cardiovasculares. Sendo o Brasil um país de dimensões continentais e elevados níveis de desigualdade, reconhecer a importância dos mecanismos de avaliação epidemiológica disponíveis no Sistema Único de Saúde (DATASUS, SIM etc.), Instituto Brasileiro de Geografia e Estatística (censo, intercenso e projeções), Programa das Nações Unidas para o Desenvolvimento (IDH, IDHM etc.), entre outros, passa a ser fundamental para assim poder direcionar políticas socio-sanitárias que orientem a aplicação de evidência científica robusta disponível e atualizada recentemente para diagnóstico, tratamento e prevenção da insuficiência cardíaca e da saúde cardiovascular em geral.^11,12^
