# Supplementary material for: Indicadores Socioeconômicos e Mortalidade por Insuficiência Cardíaca: Parâmetros Indissociáveis?
Source: Arq Bras Cardiol. 2021 Nov 1;117(5):952–3. [Article in Portuguese] doi: 10.36660/abc.20210826 (PMC8682106; doi:10.36660/abc.20210826)
Supplement: Translation

# Translation: Socioeconomic Indicators and Mortality from Heart Failure: Inseparable Parameters?

**Affiliations:** 1 Jundiaí SP Brazil Group of Investigation on Multimorbidity and Mental Health in Aging (GIMMA) - Faculdade de Medicina de Jundiaí, Jundiaí, SP - Brazil; 2 São Paulo SP Brazil Hospital Samaritano de São Paulo, São Paulo, SP - Brazil; 3 Recife PE Brazil PROCAPE (Pronto Socorro Cardiológico de Pernambuco) - Universidade de Pernambuco, Recife, PE - Brazil; 4 Recife PE Brazil Centro Cardiológico Ovídio Montenegro, Recife, PE - Brazil

**Keywords:** Cardiovascular Diseases, Heart Failure, Mortality, Risk Factor, Social Class, Human Development

Cardiovascular diseases (CVD) are still the leading cause of death, accounting for approximately one third of deaths worldwide. In June 2021, the World Health Organization (WHO) underscored its concern about the impact caused by CVD in low- and middle-income countries, where more than three quarters of their deaths occur.^1^ Heart failure (HF) is a common final route of heart diseases. It is an out-of-control global pandemic, with increasing prevalence, as a consequence of factors such as population aging, a greater presence of cardiovascular risk factors such as obesity, sedentary lifestyle or diabetes mellitus, despite therapeutic advances that reduce mortality.^2^

The connection between worse socioeconomic conditions and higher mortality from HF seems to have been well established in recent years in different populations^3–5^ and is partially justified by the worse access to diagnostic methods and pharmacological treatment. However, this relationship is more confusing in low- and middle-income countries, where clinical, demographic, and socioeconomic variables explain little about the variability between one-year HF mortality rates across Africa, India, Southeast Asia, Middle East, South America and China, as observed in the INTER-CHF prospective cohort study.^6^

In the past decades, Brazil has shown a gradual decline in inequality, measured by the Gini coefficient — especially from the mid-1990s and reaching its lowest levels in 2010^7^ — as well as a progressive improvement in the Human Development Index (HDI) and its equivalent locally determined index (LHDI), which report three basic dimensions of human development: longevity, education and income.^8^ Along the same lines, the publication by Malta et al.^9^ presented recent data confirming that the adjusted cardiovascular mortality rate has also declined in Brazil over the past years, although a heterogeneity among the states of Brazil has already drawn attention. A study published in this issue of ABC^10^ analyzes the relationship between the temporal evolution of human development and mortality rates from heart failure in different regions of Brazil, shedding some important light on this topic.

According to this study,^10^ reduced mortality from HF actually occurred in all states of Brazil. However, although the reduction in mortality in the states where there was a smaller increase in the LHDI (Rio de Janeiro, Brasília, São Paulo, Rio Grande do Sul, Santa Catarina and Espírito Santo) was greater, all of these states already had a high LHDI (>0.7). On the other hand, the authors note that the LHDI has also improved in all Brazilian states. Despite the rates lower than 0.7, the states that showed the highest increases in the LHDI (Tocantins, Maranhão, Piauí, Paraíba, Alagoas and Bahia), had smaller reductions in mortality from HF. These data strongly suggest, therefore, that to achieve large reductions in the HF mortality rate, “more important than the level of LHDI increase is the final level it reaches” – as stated by the authors.

Apparently, mortality from a chronic non-contagious disease such as heart failure and socioeconomic indicators are not such inconsistent parameters. On the contrary, it may be that these two lines meet over time in case of a reduction in inequalities and all regions reach good development rates (LHDI >0.7). Or these lines may stand apart even further if the worsening of health indicators in Brazil observed in the recent years persists, with increasing poverty rates, cuts in social policies and freezing of health investment produced by Constitutional Amendment no. 95, as recently cited.^9,11^ Although the HDI represents only a partial view of the socioeconomic status of a population, and cannot be directly assessed on the relationship of inequality and mortality due to HF, it is reasonable to infer that important variations in the HDI between regions reveal spots of inequality across Brazil. A low HDI reflects, in most cases, a poor population with a significant educational deficit, which leads to greater difficulties in understanding, acquiring and sticking to such a complex medical treatment such as HF.

The study confirms the impression that good socioeconomic and educational conditions seem to be intrinsically linked to better cardiovascular outcomes. As Brazil is a country with continental dimensions and high levels of inequality, recognizing the importance of the epidemiological assessment mechanisms available in its public health system (DATASUS, SIM, etc.), the Brazilian Institute of Geography and Statistics (census, intercensus and projections), the United Nations Development Program (HDI, LHDI, etc.), and others, is essential to direct socio-sanitary policies for the application of robust scientific evidence available and updated recently for the diagnosis, treatment and prevention of heart failure and cardiovascular health in general.^11,12^
